# An open-label Phase 2a study to assess the safety and tolerability of trimetazidine in patients with amyotrophic lateral sclerosis

**DOI:** 10.1093/braincomms/fcaf063

**Published:** 2025-02-08

**Authors:** Ruben P A van Eijk, Frederik J Steyn, Mark R Janse van Mantgem, Angela Schmidt, Myrte Meyjes, Sally Allen, Dara V Daygon, Jean-Philippe Loeffler, Ammar Al-Chalabi, Leonard H van den Berg, Robert D Henderson, Shyuan T Ngo

**Affiliations:** Department of Neurology, UMC Utrecht Brain Center, University Medical Center Utrecht, 3584 CX Utrecht, The Netherlands; Biostatistics and Research Support, Julius Center for Health Sciences and Primary Care, University Medical Center Utrecht, 3584 CX Utrecht, The Netherlands; School of Biomedical Sciences, Faculty of Medicine, The University of Queensland, St Lucia, Brisbane 4072, Australia; Department of Neurology, Royal Brisbane and Women’s Hospital, Herston, Brisbane 4006, Australia; Department of Neurology, UMC Utrecht Brain Center, University Medical Center Utrecht, 3584 CX Utrecht, The Netherlands; Australian Institute for Bioengineering and Nanotechnology, The University of Queensland, St Lucia, Brisbane 4072, Australia; Department of Neurology, UMC Utrecht Brain Center, University Medical Center Utrecht, 3584 CX Utrecht, The Netherlands; Australian Institute for Bioengineering and Nanotechnology, The University of Queensland, St Lucia, Brisbane 4072, Australia; Australian Institute for Bioengineering and Nanotechnology, The University of Queensland, St Lucia, Brisbane 4072, Australia; Queensland Metabolomics and Proteomics Facility, The University of Queensland, St Lucia, Brisbane 4072, Australia; Centre de Recherche de Biomédecine de Strasbourg (CRBS), Université de Strasbourg, 67000 Strasbourg, France; INSERM, U1118, Central and Peripheral Mechanisms of Neurodegeneration, 67085 Strasbourg, France; Maurice Wohl Clinical Neuroscience Institute, Department of Basic and Clinical Neuroscience, King's College London, London SE5 9RX, UK; Department of Neurology, UMC Utrecht Brain Center, University Medical Center Utrecht, 3584 CX Utrecht, The Netherlands; Department of Neurology, Royal Brisbane and Women’s Hospital, Herston, Brisbane 4006, Australia; Department of Neurology, Royal Brisbane and Women’s Hospital, Herston, Brisbane 4006, Australia; Australian Institute for Bioengineering and Nanotechnology, The University of Queensland, St Lucia, Brisbane 4072, Australia

**Keywords:** amyotrophic lateral sclerosis, trimetazidine, safety and tolerability, oxidative stress, hypermetabolism

## Abstract

Metabolic imbalance is associated with amyotrophic lateral sclerosis progression. Impaired glucose oxidation and increased reliance on fatty acid oxidation contribute to reduced metabolic flexibility and faster disease progression in amyotrophic lateral sclerosis. We sought to evaluate the safety and tolerability, and explore the pharmacodynamic response of trimetazidine, a partial fatty acid oxidation inhibitor, on oxidative stress markers and energy expenditure in amyotrophic lateral sclerosis. The study was conducted between June 29, 2021 and May 24, 2023. People living with amyotrophic lateral sclerosis, recruited in Australia and the Netherlands, received open-label oral trimetazidine for 12 weeks after an initial 4-week lead-in period. The primary outcome measures were safety and tolerability, as well as the change from baseline in oxidative stress markers malondialdehyde (MDA) and 8-hydroxy-2′-deoxyguanosine (8-OHdG). Secondary outcome measures were change from baseline in energy expenditure, amyotrophic lateral sclerosis functional rating scale—revised, and slow vital capacity (SVC). Linear mixed effects were used to estimate the mean difference in MDA and 8-OHdG between the on- and off-treatment periods. This trial is registered under ClinicalTrial.gov National Clinical Trial (NCT) number NCT04788745 and European Union Drug Regulating Authorities Clinical Trials (EudraCT) number 2020-005018-17. Twenty-one participants received trimetazidine; 19 (90%) completed the treatment period. Trimetazidine was well tolerated; there were 57 adverse events reported, of which 7 (11%) were deemed potentially drug-related, including hot flushes (2), nausea (2), paraesthesia (2) and fatigue (1). MDA was numerically lower during treatment [−0.29 uM; 95% confidence interval (CI) −0.90 to 0.33, *P*  *=* 0.36]; 8-OHdG was significantly lower during treatment (−0.12 nM; 95% CI −0.23 to −0.01, *P* = 0.0245). The decrease in oxidative stress markers was accompanied by a reduction in resting energy expenditure (95 kcal, 95% CI 36.8–154, *P* = 0.0014). The absence of a placebo group prevented the interpretation of the clinical parameters. Oral trimetazidine was safe and well tolerated among patients with amyotrophic lateral sclerosis. This, combined with the significant reduction in markers of oxidative stress and resting energy expenditure, warrants a larger double-blind placebo-controlled efficacy study.

## Teaser

van Eijk and Steyn *et al*. report that oral trimetazidine is safe and well tolerated in amyotrophic lateral sclerosis patients, significantly reducing oxidative stress markers and resting energy expenditure. These findings highlight the potential of trimetazidine as a therapeutic intervention for amyotrophic lateral sclerosis, warranting larger, placebo-controlled studies to confirm efficacy.

## Graphical Abstract

**Graphical Abstract:**
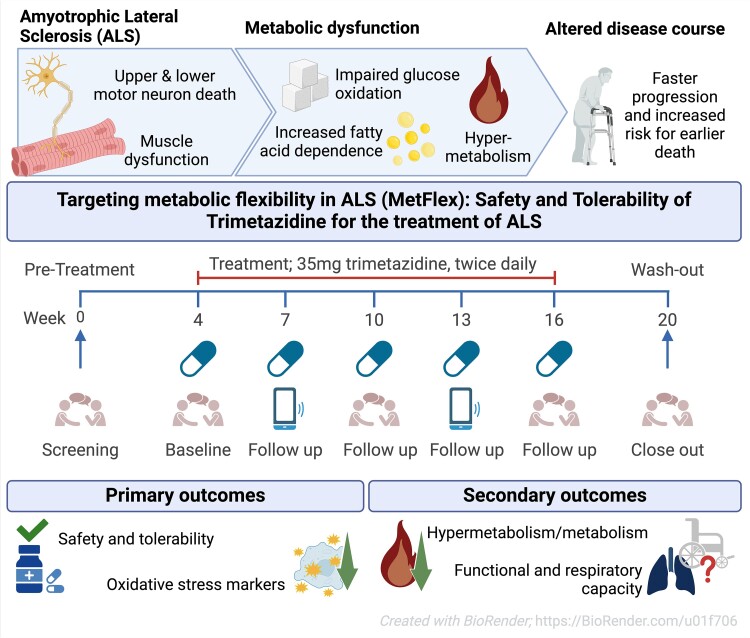

## Introduction

Amyotrophic lateral sclerosis is a complex neurodegenerative disease that is characterized by the degeneration of upper and lower motor neurons in the brain and spinal cord.^1^ While several mechanisms are proposed to underpin disease pathology, an increasing number of studies emphasize the detrimental impact of metabolic imbalances—such as hypermetabolism, impaired glucose oxidation and altered lipid metabolism—on disease progression and survival in amyotrophic lateral sclerosis.^2^ In 2018, a case-control study highlighted the clinical significance of hypermetabolism in amyotrophic lateral sclerosis, demonstrating that hypermetabolic patients have faster progression of disability and increased risk for earlier death.^3^ Hypermetabolism has now been replicated as a negative prognostic factor in amyotrophic lateral sclerosis in several geographically distinct patient cohorts, including studies from France,^4^ China^5^ and Japan.^6^ The origin of hypermetabolism in amyotrophic lateral sclerosis remains unknown; however, metabolic perturbations in skeletal muscle may be a determining factor, including an increase in the expression of pyruvate dehydrogenase kinase 4 (PDK4), which plays a central role in regulating the oxidation of glucose.^7^

In preclinical studies, there are reports of hypermetabolism in SOD1^G86R^ and SOD1^G93A^ mouse models of amyotrophic lateral sclerosis,^8-11^ and an upregulation in *Pdk4* in skeletal muscle of SOD1^G86R^ mice.^7^ Overall, the increase in *Pdk4* is proposed to lead to the inhibition of pyruvate dehydrogenase, decreased glucose oxidation and a subsequent increase in the oxidation of fatty acids for use as an alternate energy substrate—a process commonly referred to as metabolic flexibility.^7^ Indeed, in skeletal muscle of SOD1^G93A^ mice, and in primary muscle fibres grown from human amyotrophic lateral sclerosis muscle biopsies, there is a higher dependence and capacity to use fatty acids as a fuel substrate.^11^ Moreover, dysregulation of muscle cholesterol transport has recently been identified as a contributing factor in amyotrophic lateral sclerosis, further underscoring the potential role for altered lipid metabolism in disease progression.^12^ Importantly, preclinical studies show that modulation of metabolic flexibility, through promoting glucose oxidation over fatty acid oxidation, is a viable therapeutic approach in amyotrophic lateral sclerosis.^7,9,10^

Trimetazidine is a small molecule piperazine derivative that is a partial inhibitor of fatty acid oxidation approved in Europe for stable angina pectoris.^13^ By inhibiting the mitochondrial long-chain 3-ketoacyl coenzyme-A thiolase, trimetazidine evokes a shift from fatty acid to glucose utilization.^14^ Trimetazidine is also able to increase the activity of pyruvate dehydrogenase,^15^ thereby influencing energy production through the citric acid cycle. In chronic heart failure, trimetazidine significantly reduces energy expenditure (i.e. hypermetabolism)^16^ and the expression of oxidative stress markers that arise from the oxidative degradation of lipids, malondialdehyde (MDA) and 8-hydroxy-2′-deoxyguanosine (8-OHdG).^13^ Both MDA and 8-OHdG are oxidative stress markers, with some suggestive evidence that these markers are increased in people living with amyotrophic lateral sclerosis.^17^ Given the emerging evidence for the associations between oxidative stress, metabolism and amyotrophic lateral sclerosis, trimetazidine may positively affect disease progression in amyotrophic lateral sclerosis. We therefore sought to evaluate the safety and tolerability and pharmacodynamic effect of trimetazidine on oxidative stress markers and whole-body metabolism in patients with amyotrophic lateral sclerosis.

## Materials and methods

### Study design

This Phase 2a, open-label study to test the safety, tolerability and pharmacodynamic response of trimetazidine in people living with amyotrophic lateral sclerosis was conducted between 29 June 2021 and 24 May 2023. Patients were recruited from Royal Brisbane and Women’s Hospital, Brisbane, Australia, and University Medical Centre Utrecht (UMCU), Utrecht, the Netherlands. The total duration of the study was 20 weeks, consisting of a 4-week lead-in period, a 12-week on-treatment period and a 4-week washout period. The trial was conducted in accordance with the Declaration of Helsinki and the International Committee on Harmonization of Good Clinical Practice guidelines. The study was approved by the local ethics committees at Royal Brisbane and Women’s Hospital (2021/QRBW/69227) and University Medical Centre Utrecht (21/232). All participants provided written informed consent. The trial was registered under ClinicalTrial.gov National Clinical Trial (NCT) number NCT04788745 and European Union Drug Regulating Authorities Clinical Trials (EudraCT) number 2020-005018-17.

### Study population

Eligible patients had familial or sporadic amyotrophic lateral sclerosis and a diagnosis of possible, laboratory-supported probable, probable or definite amyotrophic lateral sclerosis as defined by the revised El Escorial criteria. All patients were aged between 18 and 75 years and had a Treatment Research Initiative to Cure ALS (TRICALS) risk profile between −6.0 and −2.0 (both inclusive). A TRICALS risk profile < −6.0 indicates long survival and slow disease progression, while a profile > −2.0 indicates short survival and rapid progression; ∼75% of patients fall within −6.0 and −2.0.^18^ The clinical variables to calculate the TRICALS risk score were defined according to the European Network To Cure ALS Core Clinical Dataset.^19^ The use of riluzole was permitted during the study; individuals taking riluzole were required to be on a stable dose for at least 30 days prior to the baseline visit or have stopped taking riluzole at least 30 days prior to the baseline visit. Patients were excluded from the study if they had a history of or current diagnosis of a medical condition that impacted whole-body energy metabolism (e.g. diabetes, Hashimoto’s, heart disease and hypercholesterolemia), had Parkinson’s disease or parkinsonism, had significant neuromuscular disease other than amyotrophic lateral sclerosis, had ongoing disease that may cause neuropathy, were pregnant or breastfeeding, were taking antihypertensives as trimetazidine may cause hypotension, had allergies towards one of the product’s active pharmaceutical ingredients or excipients, or had tracheostomy or non-invasive ventilation (NIV) use >22 h/day.

### Study medication

Each trimetazidine prolonged-release tablet contains 35 mg of trimetazidine dihydrochloride; the dose was based on the approved dose for stable angina pectoris. Patients received one tablet of 35 mg of trimetazidine twice daily with a glass of water during meals, except for the first dose. No specific dietary instructions were provided. The first dose was administered in the clinic on Day 1 after the 4-week lead-in period. Patients remained on treatment for 12 weeks, followed by a 4-week washout period. If a dose was not able to be taken for any reason, patients were instructed to skip the dose. If doses were missed or skipped, patients were asked to not replace them, but rather, take the next dose at the regular schedule and in the usual amount.

### Outcome measures

The co-primary outcome measures of the study were safety and tolerability, as well as the change from baseline in oxidative stress markers MDA and 8-OHdG after study drug initiation. Safety was based on safety assessments including physical examinations, clinical laboratory evaluations, vital signs and frequency of adverse events (AEs) or serious AEs (SAEs) throughout the duration of the trial. AEs and SAEs were categorized according to the Common Terminology Criteria for Adverse Events and were rated for severity and association with the study drug. Tolerability was defined as time to discontinuation of assigned treatment over the 12-week on-treatment period. Secondary outcome measures for the study were change from baseline in resting energy expenditure (REE) and the metabolic index. Exploratory measures were the change in amyotrophic lateral sclerosis functional rating scale—revised (ALSFRS-R) and % predicted SVC according to the 2012 Global Lung Initiative reference values. Plasma neurofilament light chain (NfL) levels were added as a *post hoc* measure after the trial was completed; this outcome was not originally listed in the protocol. Patients visited the clinic at Weeks −4, 0, 6, 12 and 16, during which the metabolic assessments were performed, and a blood sample was collected with the ALSFRS-R and SVC. At Weeks 3 and 9, patients were contacted by phone to assess the occurrence of AEs and obtain the ALSFRS-R. Patients who could no longer visit the clinic were offered remote follow-up by phone for AEs and the ALSFRS-R only.

### Assessment of MDA, 8-OHdG and NfL

Total MDA, a by-product of lipid peroxidation, and 8-OHdG, a biomarker for oxidative stress and DNA damage, were analysed in plasma samples by LC-MS/MS. NfL was measured in plasma samples using the NEFL assay (OID05206) from the Olink Proteomics Neuro Exploratory assay. Detailed methods are provided in the Supplementary material and Supplementary Table 1.

### Assessment of energy expenditure

Whole-body energy expenditure was assessed using an established protocol.^3^ Participants were asked to abstain from physical activity and to fast except for water for 12 h prior to the assessment at 8 am. Height was measured using a stadiometer, and body composition (fat mass and fat-free mass) was determined using a BodPod system (Cosmed USA Inc., Rome, ITA). Values of fat mass and fat-free mass were used to predict REE (predicted REE).^3^ After body composition analysis, participants underwent indirect calorimetry (Quark RM respirometer, Cosmed) on an examination bed in a semi-supine position at a 35° angle. Participants were given 10 min to relax prior to the measurement of REE (measured REE). A canopy hood was placed over the participant’s head, and the participant was allowed to rest under the hood for 5 min before the start of data collection. The ventilatory hood pump was adjusted to achieve a flow rate between 0.8 and 1.1 L/min (allowing an ∼1% FeCO_2_). Once achieved, the flow rate was not adjusted throughout the ensuing 15-min data collection period. The metabolic index, expressed as percentage deviation from the expected REE, was calculated as predicted REE (adjusted for fat-free mass, BodPod) divided by the measured REE (Quark) times 100.

### Sample size

The co-primary objectives of the study were to show a pharmacodynamic response in MDA and 8-OHdG after 12 weeks of trimetazidine treatment. At the time of study initiation, no data were available for these markers in amyotrophic lateral sclerosis. Hence, we used a related marker, namely interleukin-6, to outline a hypothesized effect size of trimetazidine. Based on a previous study,^17^ we expect a mean baseline level of interleukin-6 of 35.0 pg/mL in patients with amyotrophic lateral sclerosis. Prior work in acute pancreatitis and coronary heart disease indicated a reduction after trimetazidine treatment in interleukin-6 towards normal levels^20,21^ (9.0^17^ to 20.0^22^). We assumed conservatively a reduction of 20.0 pg/mL in interleukin-6 levels during the treatment period (from 35.0 to 15.0 with a SD of 20), resulting in a standardized effect size of 1.0. Assuming a similar effect size for MDA and 8-OHdG, thus a difference between off and on treatments of ∼1 SD, 16 patients would be required to detect such an effect using a paired Student’s *t*-test with 90% power and a two-sided alpha of 0.025 (adjusted for multiplicity). This sample size would yield ∼69% power when the effect size is 0.75 and 98% power when the effect size is 1.25. To account for missing data, we aimed to enrol 20 patients.

### Statistical analysis

Demographic data are presented using descriptive statistics (mean and standard deviation/median and interquartile range/frequency and percentage). All patients who received at least one dose of trimetazidine were included in the analysis. SAEs and AEs were categorized by system organ class according to the standardized format of the Medical Dictionary of Regulatory Activities. (S)AEs were reported as *n* (%), as number of times reported and as a rate, with the frequency of the AE being the numerator and total patient-years the denominator.

The change in MDA, 8-OHdG, NfL, measured REE and metabolic index compared with baseline (treatment Day 1) were assessed using linear mixed effects models (LMEs). The analysis incorporated all available patient data from the lead-in, on-treatment and washout periods, irrespective of dropout and drug adherence. Incomplete observations are directly addressed by the model; patients with fewer observations receive thereby less ‘weight’ in the model estimates. The model included a fixed term for a time-varying indicator variable for treatment (being 0 during lead-in and washout and 1 during on treatment) and a term for months since treatment Day 1 to account for possible time changes. The random effects included a random intercept and slope for time per patient. The time-varying coefficient of treatment was the primary estimate of the study, reflecting the mean difference in the change from baseline when on or off treatment. Significance was based on the likelihood ratio test with one degree of freedom; 95% confidence intervals (CIs) were based on the profile likelihood. For illustrative purposes, we also estimated the mean outcome level at each visit using a mixed model for repeated measures with solely a fixed effect for visit and an unstructured covariance structure to model the within-patient variances.

The average population change over time in the ALSFRS-R total score, % predicted SVC and weight were summarized with a linear mixed effects model including a fixed effect for time and a random intercept and slope for time per patient. For the analysis of the two co-primary outcomes, a *P*-value < 0.025 was considered statistically significant; the other analyses were considered exploratory. All analyses were conducted in R using the *nlme* (version 3.1-163; 2023) and *mmrm* (version 0.3.10; 2024) libraries.

## Results

**Figure 1:**
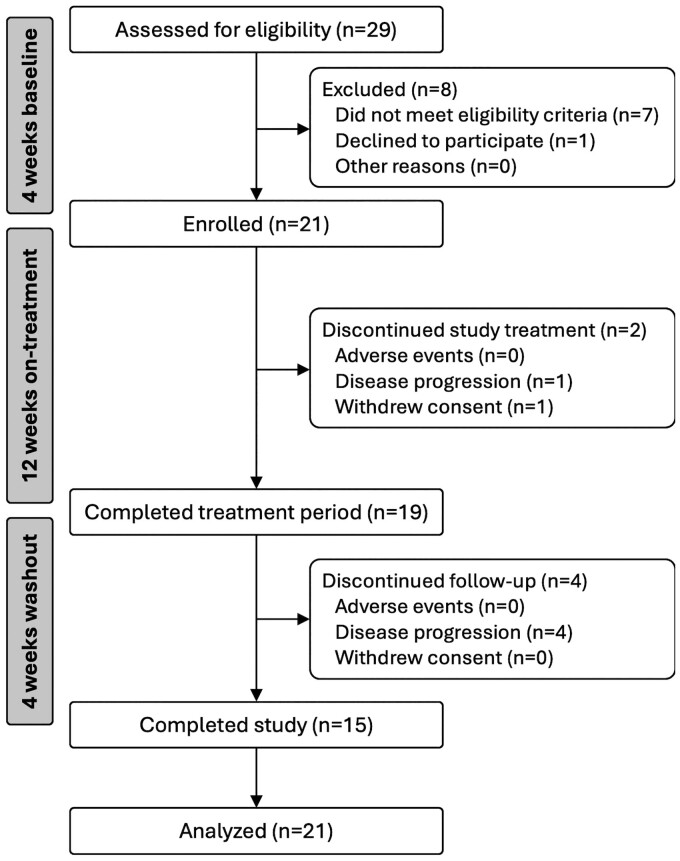
**Study flow chart.** Patients were recruited from the Royal Brisbane and Women’s Hospital (Brisbane, Australia) and University Medical Centre Utrecht (Utrecht, the Netherlands).

Twenty-nine patients were screened between June 2021 and January 2023; seven patients did not meet the inclusion criteria and one patient withdrew consent prior to receiving any study medication. Twenty-one patients were enrolled; their baseline characteristics are listed in Table 1. Of the 21 patients who received at least one dose of study treatment, six patients (29%) stopped the study prematurely: two patients stopped taking study medication [one due to disease progression and one due to withdrawal of consent (to participate in an alternate trial)], and four patients completed the 12-week study medication period but were unable to complete in-clinic visits during the 4-week post-treatment follow-up (Fig. 1).

**Table 1:** Overview of patient characteristics at baseline

Characteristic	All patients
	(*N* = 21)
Age, years	56.1 (8.9)
Sex, male	15 (71%)
Site of symptom onset, bulbar	6 (29%)
Symptom duration,^a^ months	19 (15)
El Escorial category	
Definite	5 (24%)
Probable	9 (43%)
Probable lab sup.	6 (29%)
Possible	1 (5%)
ALSFRS-R total score	35.0 (6.94)
ΔFRS,^a^ points per month	−0.55 (0.76)
% predicted SVC	85.1 (26.5)
Riluzole use	15 (71%)
TRICALS Risk Profile	−4.20 (1.26)
MDA, uM	8.25 (1.98)
8-OHdG, nM	0.80 (0.63)
Neurofilament light chain, NPX	5.33 (1.18)
REE, kcal	1806 (256)
Metabolic index, % predicted	122 (13)

Data are in mean (SD) or frequency (%).

8-OHdG, 8-hydroxy-2′-deoxyguanosine; ALSFRS-R, Amyotrophic lateral sclerosis functional rating scale—revised; kcal, kilocalories; MDA, malondialdehyde; metabolic index, predicted REE divided by the measured REE times 100; NPX, normalized protein expression using Olink Proteomics’ arbitrary unit on log2 scale; REE, resting energy expenditure; SVC, slow vital capacity; TRICALS, Treatment Research Initiative to Cure ALS; ΔFRS, decline in the ALSFRS-R (points/month since date of symptom onset).

^a^Data are in median (IQR).

### Safety, tolerability and compliance

Overall, trimetazidine was well tolerated, and 19 (90%) patients completed the 12-week treatment period; none of the patients stopped the study medication due to AEs. There were three SAEs in two patients [two an elective surgery for feeding tube insertion and once an unplanned hospitalization due to coronavirus disease-related shortness of breath]. No deaths occurred during study follow-up. There were 57 AEs reported among 18 patients during the on-treatment and washout periods; the occurrence of AEs is presented in Table 2. Of all AEs, seven (11%) were deemed possibly or probably related to trimetazidine, including the reporting of hot flushes (two), nausea (two), paraesthesia in the feet (two) and fatigue (one). All were self-limiting and disappeared after cessation of study medication. There were no statistically significant increases during the treatment period in laboratory parameters, including QT interval (start of the Q wave to the end of the T wave), kidney and liver function or increases in Parkinson-related symptoms (worsening of tremors, akinesia and hypertonia).

**Table 2:** AEs during the 12-week on-treatment and washout period (*N* = 21)

System organ class	No. of patients (%)	Total frequency	Rate
Any adverse event	18 (86%)	57	9.04
Ear and labyrinth disorders	2 (10%)	2	0.32
Fall	5 (24%)	11	1.75
Fatigue	1 (5%)	1	0.16
Gastrointestinal disorders	5 (24%)	8	1.27
General disorders and administration site conditions	3 (14%)	4	0.63
Infections and infestations	7 (33%)	9	1.43
Musculoskeletal and connective tissue disorders	4 (19%)	9	1.43
Nervous system disorders	6 (29%)	7	1.11
Psychiatric disorders	1 (5%)	1	0.16
Respiratory, thoracic and mediastinal disorders	2 (10%)	2	0.32
Skin and subcutaneous tissue disorders	3 (14%)	3	0.48

Number of patients (%). AEs were classified according to the Medical Dictionary for Regulatory Activities Terminology. The rate—in number of AEs per person-year—was calculated as the total frequency of AEs divided by the summed follow-up time across all patients during the on-treatment and washout period. The total follow-up time was 6.30 person-years.

### Change in oxidative stress markers, energy expenditure and clinical parameters

**Figure 2:**
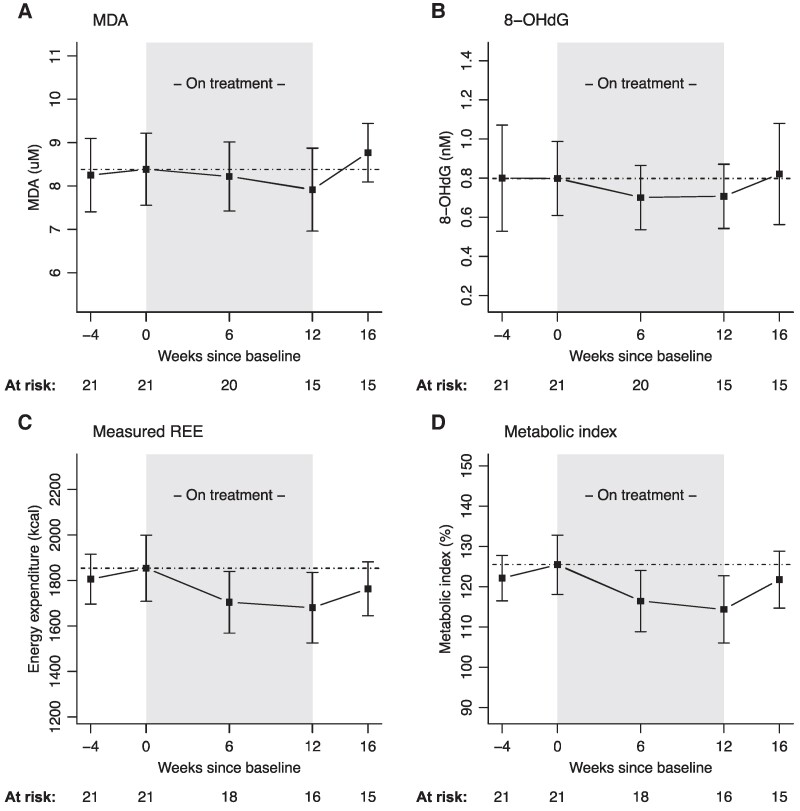
**Change from baseline in MDA, 8-OHdG and metabolic parameters.** Mixed model for repeated measures of the two co-primary biomarker concentrations (**A**) MDA and (**B**) 8-OHdG and metabolic parameters the (**C**) measured REE and (**D**) metabolic index (*N* = 21). Grey shaded area is the on-treatment period with trimetazidine; the dashed dotted line reflects the outcome level at baseline (Week 0). The error bars reflect the 95% CI around the mean. The number at risk is the number of patients with an assessment available at each time point. Averaged over on and off treatments, the mean difference for MDA was −0.29 uM (95% CI −0.90 to 0.33, *P =* 0.36), 8-OHdG was −0.12 nM (95% CI −0.23 to −0.01, *P* = 0.0245), measured REE was −95.4 kcal (95% CI −154.0 to −36.8, *P =* 0.0014), and metabolic index was −7.01% (95% CI −11.03 to −2.98, *P =* 0.0007). 8-OHdG, 8-hydroxy-2′-deoxyguanosine; kcal, kilocalories; MDA, malondialdehyde; metabolic index, predicted REE divided by the measured REE times 100; REE, resting energy expenditure.

The changes in oxidative stress markers and energy expenditure are presented in Table 3. The co-primary endpoint, MDA, was numerically lower during treatment, with a mean difference compared with off-treatment of −0.29 uM (95% CI −0.90 to 0.33, *P =* 0.36). The mean difference between on and off treatments for the co-primary endpoint 8-OHdG was statistically significantly lower during treatment, mean difference of −0.12 nM (95% CI −0.23 to −0.01, *P* = 0.0245). Interestingly, after cessation of treatment, both MDA and 8-OHdG increased (Fig. 2A and B); the individual trajectories are provided in Supplementary Fig. 1. The reduction in MDA and 8-OHdG was accompanied by a reduction in REE (*P* = 0.0014) and the metabolic index (*P* = 0.0007), which increased to baseline values after cessation of study treatment (Fig. 2C and D). NfL levels were not statistically lower during the on-treatment period compared with off-treatment period (−0.07 uM, 95% CI −0.16 to 0.04, *P* = 0.22). The ALSFRS-R declined during the study period with a monthly average of 0.32 points per month (95% 0.16–0.49, *P* < 0.001). The average monthly decline in the % predicted SVC was 0.65% (95% CI 0.38–0.93, *P* < 0.001). For weight, monthly decline was 42.1 g (95% CI −30.0 to 116.3, *P* = 0.24). The absence of a placebo group excluded the interpretation of these clinical parameters.

**Table 3:** Change from baseline in MDA, 8-OHdG and metabolic parameters

Outcome	Mean change off-treatment	Mean change on-treatment	Mean difference	95% CI	*P*-value^a^
Co-primary endpoints					
MDA (uM)	−0.03	−0.32	−0.29	−0.90 to 0.33	0.36
8-OHdG (nM)	0.00	−0.12	−0.12	−0.23 to −0.01	0.0245
Key secondary endpoints					
Measured REE (kcal)	−34.4	−129.8	−95.4	−154.0 to −36.8	0.0014
Metabolic index (%)	−2.07	−9.08	−7.01	−11.03 to −2.98	0.0007
*Post hoc* endpoint					
NfL (NPX)	0.00	−0.07	−0.06	−0.16 to 0.04	0.22

The off-treatment period comprised the 4-week lead-in period and a 4-week washout period, whereas the on-treatment period comprised the 12-week treatment period.

8-OHdG, 8-hydroxy-2′-deoxyguanosine; CI, confidence interval; kcal, kilocalories; MDA, malondialdehyde; metabolic index, predicted REE divided by the measured REE times 100; NfL, neurofilament light chain; NPX, normalised protein expression using Olink Proteomics’ arbitrary unit on log2 scale; REE, resting energy expenditure.

^a^ *P*-values and confidence intervals are not adjusted for multiplicity.

## Discussion

We sought to evaluate the safety and tolerability and pharmacodynamic effect of trimetazidine on measures of oxidative stress and whole-body metabolism in people with amyotrophic lateral sclerosis. We demonstrate that oral trimetazidine over a 12-week period is safe and well tolerated, and this is accompanied by a statistically significant reduction in measures of oxidative stress and whole-body metabolism. The short duration and absence of a randomized control group limited our ability to interpret clinical parameters. Nevertheless, given the emerging evidence for the associations between oxidative stress, metabolism, disease progression and overall survival in amyotrophic lateral sclerosis, these results warrant a larger confirmatory and efficacy study.

Overall, trimetazidine was well tolerated, with 19 (90%) patients completing the 12-week treatment period and none stopping due to AEs. There were three serious SAEs in two patients, all unrelated to the treatment. No deaths occurred during the study follow-up. Of the 57 AEs reported among 18 patients, only 7 (11%) were deemed possibly or probably related to trimetazidine, all of which were self-limiting and resolved without intervention. No significant changes in laboratory parameters, including QT interval, kidney and liver function or Parkinson-related symptoms, were observed, suggesting a favourable safety profile. While this is the first study of trimetazidine in amyotrophic lateral sclerosis, there were no discontinuations over the 12-week on-treatment period due to AEs, which aligns well with prior findings showing that trimetazidine is well tolerated in other populations.^23^ Of note, although our study population was reflective of a common trial population, older patients with relatively fast and slow progression rates were underrepresented. As such, differential safety aspects among these patients could not be evaluated in this study.

We show that markers of oxidative stress are lowered by trimetazidine, confirming the drug’s impact on its intended mechanism of action at this dose, also in people with amyotrophic lateral sclerosis. A similar effect on markers of oxidative stress was previously observed in patients with chronic heart failure.^24^ Modulation of oxidative stress has been a key therapeutic target in amyotrophic lateral sclerosis. Recently, several large efficacy trials that targeted a mechanism of oxidative stress failed to achieve clinical benefit. Studies involving edaravone,^25^ tauroursodeoxycholic acid^26^ or a combination therapy of tauroursodeoxycholic acid with sodium phenylbutyrate,^27^ while safe, showed no improvement in patient outcomes. There has been little evidence to demonstrate the lowering of oxidative stress by edaravone and tauroursodeoxycholic acid. A longitudinal study on edaravone is currently ongoing,^28^ and it remains unclear whether edaravone lowers oxidative stress in people with amyotrophic lateral sclerosis. Although no data are available for amyotrophic lateral sclerosis specifically, combination therapy of tauroursodeoxycholic acid with sodium phenylbutyrate led to an increase in oxidative stress markers (8-OHdG) in patients with Alzheimer’s disease.^29^ Overall, these therapies either work as free-radical scavengers (edaravone)^30^ or aim to mitigate endoplasmic reticulum stress and mitochondrial dysfunction,^31,32^ thereby reducing oxidative stress. Trimetazidine works through a different mechanism by directly influencing energy production through the citric acid cycle, impacting the production of reactive oxygen species and subsequent oxidative stress. Considering our positive findings, future trials that assess pharmacodynamic responses in oxidative stress markers in response to trimetazidine will cement the relevance of these reductions for amyotrophic lateral sclerosis drug development.

Findings on the impact of trimetazidine on metabolic parameters in people with amyotrophic lateral sclerosis are especially interesting. A reduction in markers of oxidative stress was accompanied by a significant decrease in REE and the metabolic index. Moreover, measures of metabolism rebounded during the washout period. This suggests that trimetazidine may help mitigate the hypermetabolic state often observed in patients with amyotrophic lateral sclerosis. Unfortunately, no data on the respiratory quotient were collected, which would have further rationalized the effect of trimetazidine on lipid oxidation. Moreover, measures of metabolic rate were based on the REE, without considering activity-induced oxygen demands that occur during the day, highlighting pertinent objectives for future investigations. Nevertheless, hypermetabolism is associated with faster disease progression and poorer prognosis,^3-6^ and previous studies that attempted to modulate metabolism, such as through high-calorie diets or the use of compounds with metabolic effects,^2^ have shown mixed results. Our findings suggest that trimetazidine, by reducing oxidative stress and normalizing metabolic rates, may have the potential to influence disease progression and related outcomes such as weight loss and energy balance in amyotrophic lateral sclerosis. As with oxidative stress markers, larger and longer duration studies are needed to confirm sustained metabolic effects, and to investigate possible therapeutic benefit, or how these pharmacodynamic changes are associated with changes in clinical parameters, and whether these markers have a broader utilization in amyotrophic lateral sclerosis beyond evaluating the response to trimetazidine.

Our focus was to evaluate the initial safety and tolerability and to determine the pharmacodynamic response of trimetazidine in people with amyotrophic lateral sclerosis. By using a lead-in and washout period, we increased the study efficiency, while also allowing assessment of on-treatment changes in pharmacodynamic biomarkers. Depending on the duration of treatment, a timely change in biomarkers after treatment initiation is required. Given the inconclusive response in MDA, the on-treatment period may have been too short, or the sample size too small to detect a disease-relevant change, if one exists. Moreover, in this study, we simply used the approved dose for angina pectoris. It remains unclear, therefore, whether dosing was appropriate and if a different dose would have resulted in more substantial reductions. Finally, the response in the oxidative stress markers may also be explained by external factors that influence metabolism, such as concomitant medication use and smoking, which cannot be completely ruled out in this uncontrolled study. Given the above limitations, a larger randomized controlled study, with multiple doses over a longer time frame, is needed to evaluate the clinical benefit of trimetazidine and its optimal dose in people living with amyotrophic lateral sclerosis. As MDA and 8-OHdG only partially reflect oxidative stress, a refined assessment is warranted with additional measurement of, for example, reactive oxygen species, antioxidant and pro-oxidant status, and the expression of genes involved in oxidative stress response to better understand the mechanisms of trimetazidine.

## Conclusion

In conclusion, we have shown that oral trimetazidine over a 12-week period is safe and well tolerated, resulting in a statistically significant reduction in markers for oxidative stress and systemic metabolic rate. Oxidative stress and metabolic rate are related to pathophysiological mechanisms in amyotrophic lateral sclerosis, and their modulation is a key target for therapeutic development. The short duration and absence of a randomized control group excluded the interpretation of clinical parameters. Nonetheless, our findings warrant a larger study that focuses on long-term effects and potential clinical benefits to fully understand the therapeutic potential of trimetazidine in amyotrophic lateral sclerosis.

## Supplementary Material

fcaf063_Supplementary_Data

## Data Availability

De-identified/anonymized data will be made available upon reasonable request by a qualified researcher.
