# Supplementary material for: An open-label Phase 2a study to assess the safety and tolerability of trimetazidine in patients with amyotrophic lateral sclerosis
Source: Brain Commun. 2025 Feb 8;7(1):fcaf063. doi: 10.1093/braincomms/fcaf063 (PMC11851067; doi:10.1093/braincomms/fcaf063)
Supplement: fcaf063_Supplementary_Data [file fcaf063_supplementary_data.docx]

**An open-label phase 2a study to assess the safety and tolerability of Trimetazidine in patients with amyotrophic lateral sclerosis**

Ruben P.A. van Eijk (0000-0002-7132-5967)^*1,2^, Frederik J. Steyn (0000-0002-4782-3608)^*3,4^, Mark R. Janse van Mantgem (0000-0002-5782-1980)^1^, Angela Schmidt^5^, Myrte Meyjes^1^, Sally Allen^5^, Dara V Daygon (0000-0003-0805-5148)^5,6^, Jean-Philippe Loeffler^7,8^, Ammar Al-Chalabi (0000-0002-4924-7712)^9^, Leonard H. van den Berg (0000-0002-5203-9674)^1^, Robert D Henderson (0000-0002-2820-81830)^4^, Shyuan T. Ngo (0000-0002-1388-2108)^4, 5^

*Ruben P.A. van Eijk and Frederik J. Steyn contributed equally to this work.

Correspondence to: A/Prof Shyuan Ngo; Australian Institute for Bioengineering and Nanotechnology; The University of Queensland, Australia 4072, s.ngo@uq.edu.au

**Author Affiliations:**

1. Department of Neurology, UMC Utrecht Brain Center, University Medical Center Utrecht, Utrecht, The Netherlands.

2. Biostatistics & Research Support, Julius Center for Health Sciences and Primary Care, University Medical Center Utrecht, Utrecht, The Netherlands.

3. School of Biomedical Sciences, Faculty of Medicine, The University of Queensland, St Lucia, Brisbane, Australia, 4072.

4. Department of Neurology, Royal Brisbane & Women’s Hospital, Brisbane, Australia, 4006.

5. Australian Institute for Bioengineering and Nanotechnology, The University of Queensland, St Lucia, Brisbane, Australia, 4072.

6. Queensland Metabolomics and Proteomics Facility, The University of Queensland, St. Lucia, Brisbane, Australia.

7. Centre de Recherche de Biomédecine de Strasbourg (CRBS), Université de Strasbourg, Strasbourg, France.

8. INSERM, U1118, Central and Peripheral Mechanisms of Neurodegeneration, Strasbourg, France.

9. Maurice Wohl Clinical Neuroscience Institute, Department of Basic and Clinical Neuroscience, King's College London, London, UK.

# **Table of Contents**

[**Table of Contents** 1](#_Toc183198628)

[**Supplementary Material** 2](#_Toc183198629)

[**Methods** 2](#_Toc183198630)

[**Supplementary Table 1. Optimised multiple reaction monitoring (MRM) transitions for LC-MS/MS analysis of 8-OHdG and MDA.** 3](#_Toc183198631)

[**Supplementary Figure 1. Trajectories of malondialdehyde (MDA) and 8-Hydroxy-2'-deoxyguanosine (8-OHdG).** 4](#_Toc183198632)

[**References:** 4](#_Toc183198633)

# **Supplementary Material**

## **Methods**

*Collection, Processing and Storage of Plasma Samples*

Blood samples were collected from the antecubital fossa of participants who were fasted for 12 hours using a winged butterfly system into a K3EDTA vacutainer. Collection of fasting blood samples minimises the impact of confounding variance in postprandial oxidative stress induction due to differences in caloric intake between individuals and visits^1^. The EDTA vacutainer containing sample was centrifuged immediately for 10 minutes at 2000 g at room temperature. The vacutainer was transferred to a decontaminated Class II Biosafety Cabinet where the supernatant plasma was pipetted into 500 uL aliquots into 1.8 mL externally-threaded screw-top Cryovials. Plasma samples were snap frozen on dry ice and transferred into an Ultra-Low (– 80 °C) Temperature Freezer with constant temperature monitoring.

*Assessment of malondialdehyde (MDA)*

Total MDA was analysed in plasma samples by hydrolysis^2^ with subsequent derivatisation to increase sensitivity and enhance detection by LC-MS/MS. Plasma (50 µl) was diluted with 1 volume water and hydrolysed with 25 µl 6M sodium hydroxide solution for 30 mins at 60°C. The solution was neutralised with 125 µl 20% trichloroacetic acid and centrifuged at 13,500 RPM at 4°C for 5 mins. 25 µl of the hydrolysed sample was spiked with 2 µM glutaraldehyde as internal standard and used for derivatisation. Derivatisation was performed using 50 µl 100mM 3-nitrophenylhydrazine in 75% acetonitrile. The solution was allowed to react for 2 hours at 42°C, then diluted 5x with 1 µM azidothymidine in water. Following derivatisation, samples were analysed on a Shimadzu Nexera UHPLC coupled to a Shimadzu 8060 LC MS-MS equipped with electrospray ionisation (ESI) operated in positive mode. Chromatographic separation was performed using a Phenomenex Gemini NX-C18 column (3 µm x 150 mm x 2 mm) with a Gemini-NX C18 guard column (4 x 2 mm). Interface and Desolvation line temperatures were set at 300 °C and 250°C, respectively. Heating gas and drying gas flow was kept at constant 10 ml/min throughout the run. The mobile phase consisted of A: 0.1% formic acid in water and B: 0.1% formic acid in acetonitrile. The separation consisted of isocratic elution of mobile phase B at 15% for 1 min, then the gradient was ramped to 98% B for 4 mins, held for another 4 mins and the column was re-equilibrated at 15% B for minutes, for a total analysis time of 11 mins. The column oven was held at 40°C.

MDA standard was synthesised as done previously^3^. Briefly, 10 µL of malondialdehyde bis(dimethyl acetal) was diluted to 10 mL in 0.1 M hydrochloric acid. The solution was boiled for 5 min in a water bath, then cooled under running water to give a final hydrolysed standard concentration of 6.07 mM. The synthesised standard solution was diluted with water from 50 µM to 40 nM. The calibration curve for MDA was linear between 200nM to 12.5uM (correlation coefficient =0.9991).

Quality control (QC) samples, calibration standards, and blank samples (water) were derivatised in the same manner as the test samples. QC samples (n=9) from a single individual volunteer were analysed every 40 samples. The precision among the QC samples, defined as the percent relative standard deviation, was 8.8%. The lower limit of quantification of the analytical method, established as the concentration of MDA giving a signal-to-noise ratio (S/N) above 10, was 200nM. All samples were analysed in duplicates.

*Assessment of 8-hydroxy-2’-deoxyguanosine (8-OHdG)*

8-OHdG was extracted from plasma samples using solid-phase extraction following previously published methods with some modifications^4,5^. 500 µl of plasma was spiked with 1 nM cordycepin as internal standard. Samples were diluted with 1 mL water and 0.01% butylated hydroxytoluene (BHT) to prevent further oxidation in the samples. Ammonium acetate (1M, 300 µl) was added to the samples before passing on to a 3cc 500mg Waters Sep-Pak tC18 SPE column, pre-equilibrated with 1 mL methanol and 1 mL water. The samples were eluted at 3-5 psi, and washed twice with 1 mL water. Finally, 8-OHdG was eluted using 800 µL 40% methanol, then 700 µL 100% methanol. The eluent was evaporated using vacuum concentration to 500 µL volume, freeze-dried and reconstituted with 60 µL 5% acetonitrile with 500 nM azidothymidine. The samples were transferred to HPLC vials for analysis.

20 µL of the extracted solution was injected into a Shimadzu Nexera UHPLC-MS/MS operated on a positive ESI mode. The mobile phase for chromatographic separation was A: 0.1% acetic acid in water; B: 0.1% acetic acid in acetonitrile. Gradient elution was performed starting at 5% B and rising to 30% B at 9 mins, before flushing the column with 100% B during the next 6 mins. Finally, the column was re-equilibrated at 5% B for another 5 mins. The interface conditions and chromatographic column were same as above. Automated peak-picking and integration was performed using Shimadzu LabSolutions insight using the internal standard method. Peak areas of the samples were calculated against calibration standards of commercial 8-OHdG reference standard (Sigma Cat. No. H5653) diluted from 10uM to 20pM. The calibration curve for 8-OHdG was linear between 300pM to 40nM (correlation coefficient =0.9999).

Quality control (QC) samples from a single individual were extracted and analysed every 20 test samples to ensure no significant variability occurred between extraction runs. Spiked quality control (SQC) samples (n=5) were prepared by adding 10nM of 8-OHdG to the quality control samples. Percent relative standard deviation among QC samples (n=21) and SQC (n=5) was calculated to be 9.84% and 4.13%, respectively. The lower limit of quantification of the analytical method, established as the concentration of 8-OHdG giving a signal-to-noise ratio (S/N) above 10, was 0.6nM. All samples were analysed in duplicates.

Scheduled multiple reaction monitoring parameters for MDA and 8-OHdG are reported in Supplementary Table 1.

*Assessment of neurofilament light chain (NfL)*

NfL levels were measured using the Proximity Extension Assay on the Olink Target platform at the Walter and Eliza Hall Institute of Medical Research (WEHI) Advanced Genomics Facility (Parkville, Australia), using the NEFL assay (OID05206) the Olink Proteomics NeuroExploratory assay. Briefly, 1µL of each plasma sample was incubated in multiplex with dual oligo-labelled detection antibodies, and proximity extension performed to create a specific DNA barcode for detection by microfluidic qPCR. CT values for NfL were then processed to NPX values by normalising to the internal Extension Control and the external Inter-plate Control using Olink Signature software and OlinkAnalyze R package (Olink Proteomics, Sweden).

## **Supplementary Table 1. Optimised multiple reaction monitoring (MRM) transitions for LC-MS/MS analysis of 8-OHdG and MDA.**

| **Compound** | **RT (min)** | **ESI mode** | **Precursor ion (m/z)** | **Product ion (m/z)** | **Dwell time (msec)** | **Q1 Pre Bias (V)** | **CE (V)** | **Q3 Pre Bias (V)** |
| --- | --- | --- | --- | --- | --- | --- | --- | --- |
| MDA-3NPH | 4.7 | + | 190.05 | 144.25 | 50.0 | -30.0 | -19.0 | -15.0 |
|  |  |  | 190.05 | 117.15 | 20.0 | -22.0 | -26.0 | -12.0 |
|  |  |  | 190.05 | 143.20 | 20.0 | -29.0 | -27.0 | -15.0 |
| GDA-3NPH | 5.0 | + | 371.05 | 218.20 | 50.0 | -37.1 | -11.1 | -25.5 |
|  |  |  | 371.05 | 175.20 | 20.0 | -19.0 | -15.7 | -19.0 |
|  |  |  | 371.05 | 82.15 | 20.0 | -19.0 | -23.0 | -15.2 |
| AZT | 3.2 | + | 268.00 | 127.00 | 25.0 | -25.3 | -19.5 | -28.2 |
|  |  |  | 268.00 | 110.10 | 25.0 | -12.3 | -30.6 | -10.8 |
|  |  |  | 268.00 | 54.00 | 25.0 | -12.3 | -46.7 | -21.0 |
| 8-OHdG | 5.5 | + | 284.00 | 168.10 | 100.0 | -25.5 | -15.1 | -19.0 |
|  |  |  | 284.00 | 140.10 | 20.0 | -13.9 | -28.8 | -13.9 |
|  |  |  | 284.00 | 112.20 | 20.0 | -26.8 | -35.5 | -20.3 |
| COR | 5.2 | + | 251.95 | 136.10 | 100.0 | -21.6 | -19.2 | -26.8 |
|  |  |  | 251.95 | 119.10 | 20.0 | -21.6 | -41.5 | -21.6 |
|  |  |  | 251.95 | 99.10 | 20.0 | -11.3 | -20.5 | -21.6 |

Abbreviations: MDA-3NPH, 3-Nitrophenylhydrazine derivative of malondialdehyde; GDA-3NPH, 3-Nitrophenylhydrazine derivative of glutaraldehyde (internal standard); AZT, azidothymidine (injection standard); 8-OHdG, 8-hydroxy-2’-deoxyguanosine; COR = cordycepin (internal standard); ESI, electrospray ionisation; RT, retention time; m/z, mass to charge ratio; V, volts.

## **Supplementary Figure 1. Trajectories of malondialdehyde (MDA) and 8-Hydroxy-2'-deoxyguanosine (8-OHdG).**

##
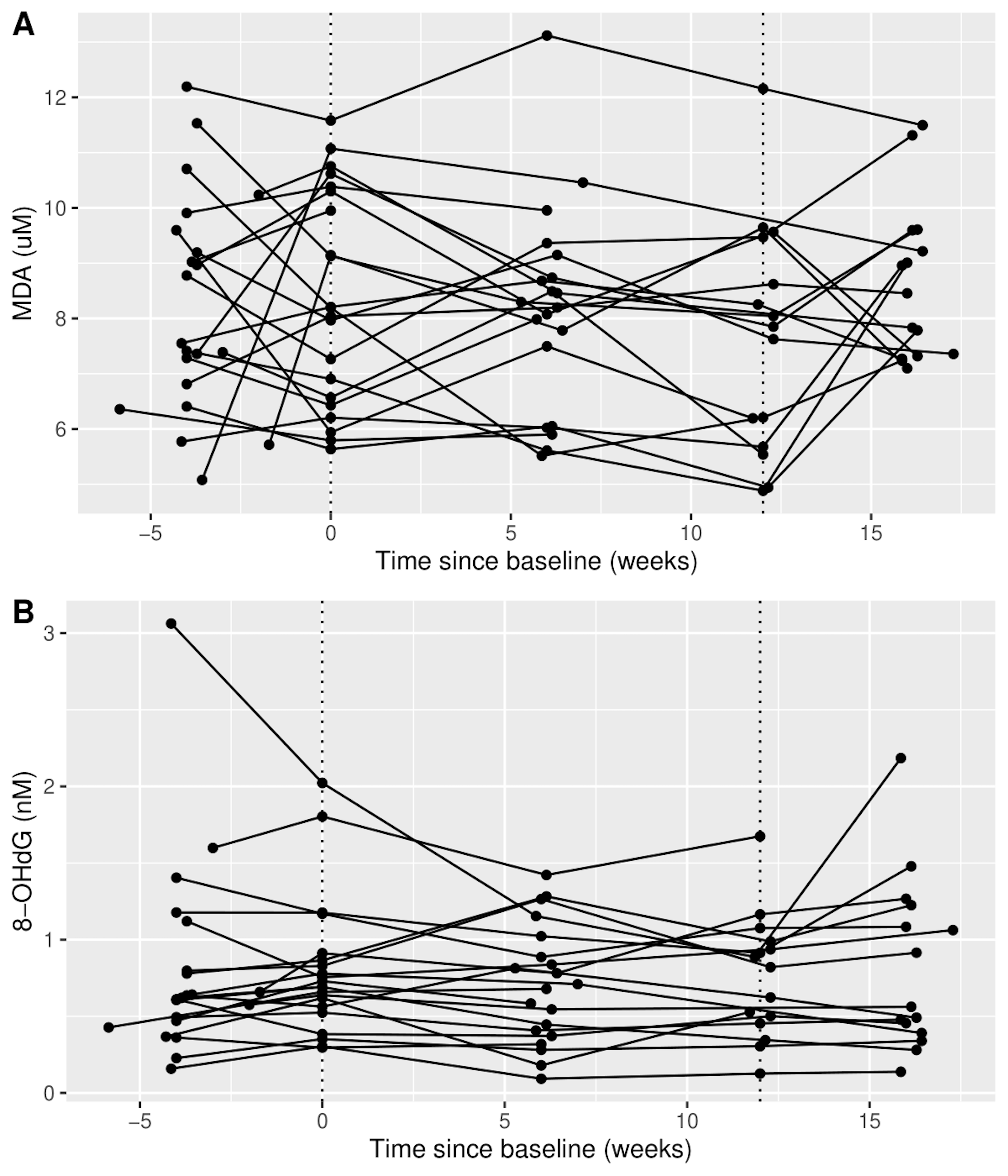


**Supplementary Figure 1. Trajectories of malondialdehyde (MDA) and 8-Hydroxy-2'-deoxyguanosine (8-OHdG).** Individual trajectories for (A) MDA and (B) 8-OHdG from screening through to close-out visit. Mixed model for repeated measures of the two co-primary biomarker concentrations (A) MDA and (B) 8-The dashed vertical lines denote treatment initiation (week 0) and cessation time points (week 12).

## **References:**

1. Gregersen, S., Samocha-Bonet, D., Heilbronn, L.K., and Campbell, L.V. (2012). Inflammatory and oxidative stress responses to high-carbohydrate and high-fat meals in healthy humans. J Nutr Metab *2012*, 238056. 10.1155/2012/238056.

2. Mendonca, R., Gning, O., Di Cesare, C., Lachat, L., Bennett, N.C., Helfenstein, F., and Glauser, G. (2017). Sensitive and selective quantification of free and total malondialdehyde in plasma using UHPLC-HRMS. J Lipid Res *58*, 1924-1931. 10.1194/jlr.D076661.

3. Kakuda, Y., Stanley, F.R., and van de Voort, F.R. (1981). Determination of TBA number by high performance liquid chromatography. JAOCS *58*, A773-A775. 10.1007/BF02887320.

4. Hu, C.W., Wu, M.T., Chao, M.R., Pan, C.H., Wang, C.J., Swenberg, J.A., and Wu, K.Y. (2004). Comparison of analyses of urinary 8-hydroxy-2'-deoxyguanosine by isotope-dilution liquid chromatography with electrospray tandem mass spectrometry and by enzyme-linked immunosorbent assay. Rapid Commun Mass Spectrom *18*, 505-510. 10.1002/rcm.1367.

5. Wang, C.C., Chen, W.L., Lin, C.M., Lai, C.H., Loh, C.H., Chen, H.I., and Liou, S.H. (2016). The relationship between plasma and urinary 8-hydroxy-2-deoxyguanosine biomarkers measured by liquid chromatography tandem mass spectrometry. Environ Sci Pollut Res Int *23*, 17496-17502. 10.1007/s11356-016-6898-4.
